# Liposome destruction by a collapsing cavitation microbubble: A numerical study

**DOI:** 10.1016/j.ultsonch.2021.105706

**Published:** 2021-08-12

**Authors:** Jure Zevnik, Matevž Dular

**Affiliations:** a University of Ljubljana, Faculty of Mechanical Engineering, Aškerčeva cesta 6, Ljubljana, Slovenia

**Keywords:** Bubble dynamics, Cavitation, Fluid-structure interaction, Shock wave emission, Giant lipid vesicles, DOPC

## Abstract

Hydrodynamic cavitation poses as a promising new method for wastewater treatment as it has been shown to be able to eradicate bacteria, inactivate viruses, and destroy other biological structures, such as liposomes. Although engineers are already commercializing devices that employ cavitation, we are still not able to answer the fundamental question: What exactly are the damaging mechanisms of hydrodynamic cavitation in various applications? In this light, the present paper numerically addresses the interaction between a single cavitation microbubble and a nearby lipid vesicle of a similar size. A coupled fluid–structure interaction model is employed, from which three critical modes of vesicle deformation are identified and temporally placed in relation to their corresponding driving mechanisms: (a) unilateral stretching at the waist of the liposome during the first bubble collapse and subsequent shock wave propagation, (b) local wrinkling at the tip until the bubble rebounds, and (c) bilateral stretching at the tip of the liposome during the phase of a second bubble contraction. Here, unilateral and bilateral stretching refer to the local in-plane extension of the bilayer in one and both principal directions, respectively. Results are discussed with respect to critical dimensionless distance for vesicle poration and rupture. Liposomes with initially equilibrated envelopes are not expected to be structurally compromised in cases with δ>1.0, when a nearby collapsing bubble is not in their direct contact. However, the critical dimensionless distance for the case of an envelope with pre-existing pores is identified at δ=1.9. Additionally, the influence of liposome-bubble size ratio is addressed, from which a higher potential of larger bubbles for causing stretching-induced liposome destruction can be identified.

## Highlights

• Cavitation microbubble collapse in vicinity of a liposome is investigated numerically.
• Non-attached microbubbles exhibit spherical behavior regardless of vesicle presence.
• Three critical modes of vesicle deformation and their driving forces are identified.
• Effective non-dimensional distances δ for liposome poration and rupture are given.
• A higher potential of larger bubbles for liposome destruction is identified.

## 1 Introduction

Cavitation is a physical phenomenon that can occur in liquids and is accompanied by the appearance of vaporous and gaseous cavities, commonly recognized as bubbles, that grow and collapse due to changes in ambient pressure. At first, cavitation was recognized solely as a nuisance, as it can cause unwanted vibrations, noise, and material erosion in hydraulic machinery [1]. Nevertheless, today cavitation is being utilized in various applications in the fields of chemistry [2], medicine [3], and environmental protection [4]. Hydrodynamic cavitation also poses as a promising new method for wastewater treatment [5], as it has been shown to be able to eradicate bacteria [6], inactivate viruses [7], and destroy other biological structures, such as liposomes [8].

Liposomes are lipid vesicles, that comprise of a thin spherical envelope and a liquid aqueous interior. Their envelope consists of at least one lipid bilayer, which is most commonly composed of phospholipids. Due to their similarity to a cell membrane, liposomes are used as artificial cells and model systems to study properties and stability of lipid bilayers [9]. In our recent study [8], we have demonstrated, that hydrodynamic cavitation is one of the most effective physico-chemical treatments for destroying giant 1,2-dioleoyl-sn-glycero-3-phosphocholine (DOPC) lipid vesicles, liposomes that measure over 1 μm in diameter and possess an envelope of a single DOPC lipid bilayer. As the effects of hydrodynamic cavitation were comparable to an ultrasound treatment, multiple questions regarding the optimization of hydrodynamic cavitation treatment for destruction of various biological structures remain. For example, different flow conditions are effective against bacteria [6] as they are against viruses [7].

Numerous potentially damaging mechanisms that accompany hydrodynamic cavitation can be speculated [10], such as strong shear flows [11], jets [12], high local temperatures [13] and pressure changes [14], shock waves [15], and formation of highly reactive free radicals [16]. In addition to this, one could also expect the bubbles to start forming and growing within the bilayer or the vesicle itself, which could lead to local bilayer poration and liposome stretching [17]. However, the contribution of the different mechanisms and their possible synergistic effects in various applications are still being explored [10]. In order to better understand the fundamental physics behind the interaction between bubbles and biological structures, such as here considered liposomes, more research concerning single bubble dynamics in vicinity of freely submerged deformable structures on a micro scale is needed. In the present case, we can distinguish between bubble-liposome interaction on three different spatial scales. Cavitation bubbles can be significantly larger (≫1μm), of a similar size, or smaller (≪1μm) than here considered vesicles (~1μm). As the smaller nano bubbles tend to exhibit a high degree of stability in regard to ambient pressure oscillations [1], we suspect micro and macro bubbles to have the greatest damage potential in the case of hydrodynamic cavitation.

In the present paper, we numerically address the former – the interaction between a single microbubble in vicinity of a DOPC lipid vesicle of a similar size, as most cavities in water have an initial diameter in the order of a few micrometers [18]. Our main interest is to determine the potentially destructive mechanical mechanisms of microbubble-liposome interaction, which would allow us to better explain the reasons behind the previously observed liposome destruction by the hydrodynamic cavitation treatment [8]. Besides giant liposomes, the chosen spatial scale in the order of 1 to 10 μm is similar to the size of a vast variety of pathogenic and potentially harmful microorganisms, such as most bacteria, certain cyanobacteria, unicellular algae, and yeast cells [19]. Additionally, viruses can reach the diameter of several hundred nanometers [19], which is still relatively close to the spatial scale considered in the present study. However, in all of the given examples, the extent of their structural similarity to DOPC vesicles has to be carefully considered if one attempts to extrapolate the findings of the present study to other biological structures.

First, we acknowledge the existing experimental and numerical research on the subject of a cavitation bubble interacting with various freely submerged structures on a micro scale. Marmottant and Hilgenfeldt [20] showed that gently oscillating single bubbles (equilibrium radius $R_{\mathrm{eq}}$ of 10–100 μm) excited by an ultrasound can already result in controlled deformation and lysis of DOPC vesicles of similar sizes. The authors conclude that the acoustic microstreaming, induced by the bubble, plays a key role in vesicle poration as it exerts large enough shear forces on vesicles in the flow to manipulate, deform, and rupture them. In their later work [21], the authors derived analytical predictions of vesicle shape progression and found two possible modes of liposome rupture: a) pore formation at the waist of the vesicle in the case of sufficiently large shear rates, which cause the local tension to exceed the rupture treshold and b) liposome buckling at the poles in the case of sufficient liposome elongation. Although the authors acknowledge that the shear flow is focused on a relatively small volume due to a rapid decay of the acoustic streaming velocity of the bubble, the effective bubble-liposome distance for its destruction is unfortunately not directly reported, as is might not be the most relevant for the case of continuous periodic bubble oscillations. This was addressed later by Zhou et al. [22], who acoustically excited single laser-produced microbubbles ($R_{\mathrm{eq}}=5–12\;\mu \text{m}$) to induce their growth and a subsequent collapse in vicinity of a Xenopus oocyte (cell radius $R_{\mathrm{cell}}\sim$ 400 μm). The authors report the effective initial bubble-cell distance for membrane poration to be $1.5R_{\mathrm{eq}}$ and the mean pore radius in the order of a tenth of a micrometer. Additionally, they observed a steep decline in membrane disturbance by increasing bubble-cell distance to $2R_{\mathrm{eq}}$ and no effect beyond $6R_{\mathrm{eq}}$. Effective distance for cell poration was later also reported by Le Gac et al. [23], who used a single laser-induced cavitation bubble (maximum bubble radius $R_{\max}\approx$ 40 μm) in a microfluidic confinement to porate suspended human promyelocytic leukemia cells ($R_{\mathrm{cell}}\sim$ 6 μm). Cell lysis probability of more than 75% was observed for cells located $⩽0.75R_{\max}$ away from the cavitation bubble center, while cells farther away than $4R_{\max}$ seemed to have been unaffected. Unfortunately, the utilized definitions of bubble-cell distance differ between both studies, which reduces the reader’s ability to directly compare the obtained results. Nevertheless, based on the results of both studies we estimate the critical bubble-cell distance to be similar between both studies. We find their consistency fairly surprising, as the former researchers considered cells much larger in comparison to cavitation bubbles ($R_{\mathrm{cell}}/R_{\mathrm{eq}}\approx 40$), whereas in the latter case their size ratio was the opposite and in the range of $R_{\mathrm{cell}}/R_{\max}\approx 0.15$. This suggests that cell poration is not largely dependent on the bubble-cell size ratio, however it may still play a role on the extent of the cell’s membrane disruption – local poration versus its complete destruction. Later, Li et al. [24] reported the poration of single myeloma cells by microbubble jetting, which, while certainly interesting, is unfortunately not directly applicable to the present study, as the reason for the asymmetric bubble collapse was the nearby cell trapping structure, rather than the presence of a myeloma cell itself.

The considered topic was also addressed numerically in the past. Most of the research is based on the potential flow theory along with boundary element method (BEM) to resolve bubble dynamics, without the consideration of viscous effects and compressibility of the surrounding fluid. Both Gracewski et al. [25] and later Guo et al. [26] considered ultrasonically excited microbubbles in vicinity of a deformable sphere and a red blood cell, respectively. In most cases results showed formation of an axial jet away from the cell and a maximum areal expansion of a cell in the order of 0.1 %, which is well below the rupture threshold of a few percent [27]. On the other hand, the experimental observations and complementary BEM-based simulations of Tandiono et al. [28] show that a single laser induced microbubble ($R_{\max}$ between 30 and 100 μm) can significantly stretch red blood cells ($R_{\mathrm{cell}}\sim$ 4 μm), up to five times their initial size. Noticeable elongation can also occur in cells deformed by a nearby acoustically actuated bubble, which can be employed to characterize cell deformability for various diagnostic and biological purposes [29]. Although viscous and compressibility effects normally play a minor role on bubble dynamics [1], they gain importance when considering micro and nano scale bubbles, strongly collapsing bubbles, emitted shock waves, and bubble’s interaction with nearby objects. Our recent numerical study [30] addressed a single collapsing microbubble ($R_{\mathrm{eq}}=1$ μm) in vicinity of a freely submerged spherical particle of a similar size. There, a different numerical approach was employed, a finite volume method (FVM) along with the volume of fluid (VOF) method to resolve compressible viscous multiphase flow. The reported results show only slight deviations of bubble shape from the initial spherical for the cases of non-attached bubbles, which indicates that the formation of a strong jet towards a submerged particle on a micro scale is highly unlikely due to the cushioning effects of surface tension and a relatively low impact of the particle’s presence on the bubble dynamics itself. In addition, the results show that although the gas inside a collapsing bubble can locally reach several thousand Kelvin, the thermal damage seems to be irrelevant in the cases where a similarly sized particle is not initially in direct contact with the collapsing bubble. This can be explained by the fact that the temperature field inside the collapsing bubble is not uniform and that the thickness of the thermal boundary layer is much smaller than the radius of the collapsing bubble. This further limits the search for the most likely mechanisms of destruction of liposomes and other biological structures during hydrodynamic cavitation treatment.

As already mentioned above, the present paper numerically addresses interaction between a single cavitation microbubble and a nearby DOPC lipid vesicle of a similar size. Temporal and spatial scale of the considered phenomenon is in the order of 10 ns and 1 μm, respectively. A coupled fluid–structure interaction (FSI) model is employed, which considers the influence of liposome’s deformability on the surrounding fluid flow and bubble dynamics, and vice versa. Compressible multiphase flow is resolved using a FVM/VOF approach, whereas the liposome’s envelope is modeled as a compliant structure through the finite element method. By choosing a continuum mechanics approach we are able to consider the system’s macroscopic properties, such as areal expansion of the bilayer, viscosity, compressibility, and surface tension of fluids, etc. Through this we omit modeling of the actual molecular dynamics on a local, nano and subnanoscale, and neglect certain phenomena such as hydrophobic attraction and hydrophilic repulsion. This can be justified by the fact that even when bubble and liposome are in direct contact, the peak magnitudes of hydrophobic attraction force are expected to not exceed a few tens of Nanonewton [31], [32], [33]. Additionally, the attraction will significantly decay after their separation of only a few nanometers, as is seems to adhere to the exponential trend with the decay length in the order of a nanometer [32], [33]. For reference, the pressure force that causes a microbubble to collapse in the present case is in the order of 0.1 mN.

As the bubble collapses due to increase in ambient pressure, vesicle deforms according to the temporal development of the surrounding flow field. An emphasis is given on various modes of vesicle’s deformation (bilayer stretching and wrinkling) and their corresponding driving mechanisms, from which effective distances for liposome poration and rupture are identified. Results are discussed with respect to vesicle destruction by the hydrodynamic cavitation treatment. Besides the effective bubble-liposome distance, the influence of their size ratio is also discussed.

## 2 Theoretical background and numerical model

In this section we present the considered physics and employed models to resolve bubble-liposome interaction. The computational domain is split into two sub-domains: a fluid domain, that consists of a gas bubble in a surrounding liquid – water, and a solid domain, that comprises of a spherical vesicle’s envelope. Both sub-domains are coupled together to form the final fluid–structure interaction (FSI) model. Since liposomes contain an aqueous core, we consider it as a compressible and viscous liquid, which is therefore numerically resolved as a part of the fluid domain. It is true that based on the purpose of liposome generation, various drugs, contrast agents, genetic material, etc., can be dissolved in the aqueous solution, which could to some degree affect some of its properties, such as viscosity. However, as this is largely dependent on a specific application, we decided to consider interior as water for the sake of generality.

### 2.1 Fluid dynamics model

The compressible multiphase flow is modeled using a finite volume method based solver [34], which was already employed by different authors to model various cases of spherical and non-spherical bubble dynamics [35], [36], [37], [30]. The volume of fluid method is used to resolve multiphase flow, where the interface between both phases, liquid and gas, is tracked by solving a single continuity equation (Eq. (1)) for the volume fraction of water $\alpha_{w}$.

(1) $$\frac{\partial \left(\alpha_{w}\rho_{w}\right)}{\partial t}+\nabla \cdot \left(\alpha_{w}\rho_{w}V_{w}\right)=0$$

Here $\rho_{w}$ and $V_{w}$ denote the density and velocity vector of the liquid phase, i.e., water. Based on the obtained volume fraction field, we can determine the volume-averaged material properties throughout the fluid domain. Following this, a single momentum (Eq. (2)) and energy (Eq. (3)) equation is solved, from which the shared velocity V and temperature *T* field is obtained based on the already determined material properties.

(2) $$\frac{\partial }{\partial t}(\rho V)+\nabla \cdot \left(\rho V⊗V\right)=-\nabla p+\nabla \cdot \tau +f$$

(3) $$\frac{\partial }{\partial t}(\rho e)+\nabla \cdot \left(V(\rho e+p)\right)=\nabla \cdot \left(k\nabla T\right)$$

Here *p* denotes pressure, f body forces, *k* thermal conductivity, and τ the viscous stress tensor that can be written for Newtonian fluids as

(4) $$\tau =\mu \left[\left(\nabla V+{\left(\nabla V\right)}^{T}\right)-\frac{2}{3}\left(\nabla \cdot V\right)I\right],$$

where μ is dynamic viscosity and I the unit tensor. Total specific energy *e* can be written as

(5) $$e=h-\frac{p}{\rho }+\frac{V^{2}}{2},$$

where *h* is specific enthalpy. Additionally, the effects of surface tension are included in the procedure with a body force in the momentum equation, according to the continuum surface force model [38]:

(6) $$F_{\mathrm{vol}}=\gamma \frac{\rho \kappa_{g}\nabla \alpha_{g}}{\frac{1}{2}\left(\rho_{g}+\rho_{w}\right)},$$

where γ is surface tension, $\alpha_{g}$ gas volume fraction field, and $\rho ,\rho_{g},\rho_{w}$ the densities of the mixture, gas, and liquid phase, respectively. $\kappa_{g}$ denotes bubble surface curvature, which is calculated as $\kappa_{g}=\nabla \cdot \frac{n}{\left|n\right|}$, where n is a bubble surface normal, obtained as a gradient of the gas volume fraction field.

A modified version of the Tait’s equation of state is employed to consider the nonlinear compressibility of water:

(7) $${\left(\frac{\rho }{\rho_{\mathrm{ref}}}\right)}^{n}=\frac{K}{K_{\mathrm{ref}}},$$

where the bulk modulus of water *K* at pressure *p* is calculated as $K=K_{\mathrm{ref}}+n(p-p_{\mathrm{ref}})$. The term *n* is the density exponent and $K_{\mathrm{ref}}$ the reference bulk modulus at the reference pressure $p_{\mathrm{ref}}$. For water, we consider the values of n=7.15, and $K_{\mathrm{ref}}=2.2$ GPa, $\rho_{\mathrm{ref}}=998.2$ kg/$m^{3}$ at $p_{\mathrm{ref}}=101325$ Pa [39]. The bubble contents are modeled as air with the ideal gas law, which states

(8) $$\rho =\frac{p}{R_{\mathrm{air}}^{*}T},$$

where a specific gas constant $R_{\mathrm{air}}^{*}$ of 287 J/kgK for dry air is considered. Through this, we neglect the bubble’s vapor content and its mass transfer mechanisms – evaporation and condensation. Although vapor pressure is small in comparison to the internal bubble pressure and therefore does not noticeably effect the bubble dynamics in the presently considered case [30], the mass transfer on the other hand could. As the bubble collapses, its contents are compressed, which results in locally elevated temperatures and pressures. In the case of strong bubble compression, i.e. a strong collapse, a fraction of its vapor contents are lost to the ambient liquid through the process of condensation. Even though this does not significantly influence bubble dynamics until the first collapse, the amount of non-condensable gas in the bubble can affect the magnitude of bubble’s rebound and its subsequent oscillations [40]. Due to the complex nature of mass transfer mechanisms, their adequate consideration remains one of the challenges up to this day. While it is true that they can be included with empirical models, this approach requires fitting of empirical model parameters to match the obtained numerical results to the experimental data for each separate case, which limits its applicability for smaller micro and nano bubbles. Even though that the present paper considers inertially collapsing bubbles, the intensity of their collapse is relatively weak ($R_{\max}/R_{\min}\sim 10$) in comparison to laser induced bubbles, where the ratio between the maximum and minimum bubble ratio can exceed one hundred. Based on all this, we see the use of ideal gas law as a fair approximation for the presently considered phenomenon.

For all calculations the Pressure-implicit with splitting of operators (PISO) pressure–velocity coupling algorithm [41] was employed, along with a first order implicit temporal discretization. Pressure staggering option (PRESTO!) scheme [42] for the spatial discretization of pressure was used, while density, momentum, and energy were discretized using the second order upwind scheme. A Piecewise linear interface calculation (PLIC) geometric reconstruction scheme [43] was used as a numerical implementation of the VOF method to capture the water-bubble interface. Boundary conditions at the end of the computational domain were set to wave non-reflecting pressure outlet, which was placed reasonably far away from the bubble ($\sim 100R_{\max}$) to minimize its influence on the bubble-liposome interaction. For a further insight into the numerical model readers are referred to [30], where the considered theoretical background and a numerical model description is given in more detail.

Additionally, we utilize a spring-based dynamic mesh smoothing method to adapt the numerical grid of the fluid domain to the bilayer’s movement during each FSI coupling iteration step. In this method the edges between mesh nodes in the fluid domain are represented as a network of linearly elastic springs that obey the Hooke’s law. Displacements of the internal mesh nodes are calculated according to the user specified spring constant factor and the obtained displacements of the lipid bilayer from the structural model.

### 2.2 Structure dynamics model

The envelope of a liposome is modeled as a thin spherical shell structure, as the ratio of bilayer thickness τ to liposome radius $R_{L}$ can be neglected in comparison with unity for giant unilamellar vesicles ($\tau /R_{L}<1/100$). Through this we are able to consider the envelope’s macroscopic properties, such as areal expansion and bending stiffness, but omit modeling of the actual molecular dynamics on a local, nano and subnanoscale. The dynamic response of a shell structure to the bubble-induced loads is resolved using a nonlinear finite element method based transient structural solver [44]. The time-varying displacements, strains, and stresses of the envelope are obtained by solving the following equation of motion

(9) $$M\overset{¨}{u}+C\overset{˙}{u}+Ku=f,$$

where M,C, and K represent the corresponding mass, damping, and stiffness matrices of the structure, respectively. f and u denote the load and nodal displacement vectors, whereas on overdot represents the derivative with respect to time. Large deflections are considered and true stresses and strains are considered in the model as vesicles are expected to exert a high level of compliance.

The displacement vector u can be obtained from u=x-X, where x and X correspond to the nodal position vectors in the deformed and undeformed state, respectively. From this the deformation gradient tensor F can be obtained as

(10) $$F=I+\frac{\partial u}{X},$$

where I denotes the identity matrix. The deformation gradient is a second-order tensor, which can be decomposed into a product of rotation R and right stretch tensor U. Logarithmic strain tensor ε (also known as true or Hencky strain) is defined as

(11) ε=lnU,

and can be calculated at the locations of the element integration points through the spectral decomposition of U.

The numerical model uses a generalized Hilber-Hughes-Taylor-α method [45] for implicit time integration and a full Newton–Raphson method in which the stiffness matrix is updated at every equilibrium iteration [46]. Both methods come as one of the standard options in the utilized structure dynamics solver [44]. The shell itself was geometrically defined with a surface through its mid-plane and was discretized with second order shell elements with four in-plane integration points (element SHELL281). Three integration points through the thickness of the shell were considered, which correspond to its mid, bottom, and top-surface. The last two are offset in the normal direction of the mid-surface for the half of the shell’s thickness (± τ/2).

### 2.3 Material model

The DOPC bilayer is modeled as a linearly elastic material with the equivalent elastic modulus of $\overset{‾}{E}=53.3$ MPa and Poisson’s ratio of ν=0.485 [47]. The considered value of elastic modulus $\overset{‾}{E}$ is obtained over the whole area of interest on the σ-ε curve, bounded by the material failure criterion of $\varepsilon_{p}^{*}$ (see Appendix A). Through this, we also indirectly account for stress-softening of the material at larger strains. The bilayer thickness $\tau_{0}$ is taken to be 4 nm in its undeformed state. Material damping and viscoelasticity in the present case are not considered since viscous dissipation in the adjacent aqueous phase dominates the dynamic response of vesicles to macroscopic shear deformations [48]. Additionally, according to Wu et al. [49] the viscoelastic relaxation parameter for giant lipid bilayer vesicles is in the order of 0.1 s, which by several orders of magnitude exceeds the duration of herein considered phenomenon. The material density of a DOPC bilayer is set to 1009 kg/$m^{3}$ [50], which is $\sim 1.01\rho_{w}$, as common for phospholipids.

Material failure and pore formation within the bilayer are not directly modeled but estimated by comparing the obtained stresses and strains in the envelope with the reported bilayer rupture thresholds from the literature. Bilayer rupture is generally considered to occur at tensions γ from 1 to 25 mN/m [51], [52], [53], which corresponds to areal strains in the order of 2 to 5%. Nevertheless, Evans et al. [54] showed that the ultimate membrane tension before its rupture is not a static material property, as it can for DOPC bilayers vary from 6 mN/m to 13 mN/m for loading rates of 0.07 mN/m/s and 25 mN/m/s, respectively. This data suggests that membrane rupture tension largely depends on the loading rate.

Additionally, the authors [54] identified two different dynamic regimes of membrane strength, a low-strength cavitation-limited and a high-strength defect-limited regime, with a transition at loading rates around 10 mN/m/s for DOPC bilayers. Due to the significantly shorter time scale of the considered phenomena in the present case (t~ 10 ns), here encountered loading rates exceed the experimentally achievable values by several orders of magnitude (present loading rate $\sim 10^{9}-10^{10}$ mN/m/s versus the peak experimental loading rates $\sim 10^{2}$ mN/m/s [54], [55]). According to the defect-limited kinetic model for membrane failure [54], the rupture strength rises logarithmically with the loading rate, and should be between 80 and 95 mN/m for the present case. To be more precise, the model predicts a critical membrane tension of 92 mN/m for a loading time of 10 ns. Although this value is obtained for a loading rate that highly surpasses the scope of experimental observations, it fits surprisingly well with the obtained value of 90 mN/m from molecular dynamics simulations for a liquid-phase DPPC bilayer [56]. The reported critical tension corresponds to the lateral membrane stress of $\sigma^{*}=20$ MPa, which was later also shown by Xie et al. [57] to result in bilayer rupture in a matter of a few nanoseconds, regardless of the loading regime. Additionally, Leontiadou et al. [56] also reported that uniform lateral loading of 5 MPa is already enough to cause unstable growth of pre-existing meta-stable pores, which could be thought of as a secondary failure criterion.

Based on these values, we consider two membrane rupture criterions (see Appendix B). The primary failure criterion is related to the creation of a defect in the case of heavy lateral loading and is set at linear strain of $\varepsilon_{p}^{*}=0.45$, whereas the secondary criterion, connected to the expansion of pre-existing pores, is identified at $\varepsilon_{s}^{*}=0.035$. The likelihood of vesicle destruction can be thus estimated by the phenomenological criterion of maximum strain, where maximum principal strain in the bilayer is compared to both material failure thresholds, $\varepsilon_{p}^{*}$ and $\varepsilon_{s}^{*}$.

### 2.4 Model coupling

Both separate solvers, the fluid and structure dynamics model, are coupled together according to the partitioned iterative approach, to form the final FSI numerical model. The utilized model coupling framework [58] was previously shown to be capable of capturing complex multiphysics problems and has already been verified and validated in various engineering applications [59]. In this manner we consider both, the influence of liposome’s deformability on the surrounding fluid flow and bubble dynamics, and vice versa, which is crucial given the strong coupling nature of the phenomenon under consideration. Two-way coupling in the FSI model is achieved trough the exchange of loads and displacements of the coupling interface. In our case, both sides of the liposome’s envelope represent the fluid–solid interface, through which incremental displacements and forces are transferred at each coupling iteration. The following coupling solution procedure is applied within each coupling iteration:

• the structural solver resolves the bilayer’s response to the recieved loads,
• incremental displacements of the bilayer are transferred from the structural to the fluid solver,
• computational mesh of the fluid domain is updated according to the received displacements,
• the fluid solver computes the corresponding solution,
• normal and shear forces acting on the bilayer are transferred back to the structural solver as external loads.

The given procedure is repeated until the desired level of data transfer convergence in reached, which is then followed by the advance of the whole system to the next time step. More precisely, root mean square convergence is monitored for both data transfers, force and incremental displacement, at both FSI participants, fluid and structural dynamics solver. The exchange of data, i.e., loads and displacements at the FSI interface, is achieved through mapping, which establishes a link between both coupling participants at the beginning of the simulation. The profile-preserving and conservative mapping procedure was used for the exchange of displacements and forces, respectively. In the former case, the mapping weights were determined through the use of shape functions, whereas in the latter case the intersect-scatter–gather algorithm was used [58].

### 2.5 Model setup

As already mentioned, we consider a single cavitation bubble of radius $R_{0}=R_{\max}=1\;\mu$m and a nearby liposome of radius $R_{L}=1\;\mu$m, both at equilibrium with an initial ambient pressure of one atmosphere $p_{\infty ,0}=101325$ Pa and an ambient temperature of 20°C. According to the Young–Laplace equation the corresponding internal bubble pressure amounts to $2.47\times 10^{5}$ Pa. Inertial bubble collapse is induced with a sudden ambient pressure increase to $p_{\infty }=10^{7}$ Pa, which is a typical value one could expect to occur on here considered spatial (~1 μm) and temporal (~10 ns) scales in the case of hydrodynamic cavitation. To further clarify, this value does not represent the operating pressure of a given hydraulic system, but rather one that can locally occur within a cavitating flow, e.g. the ambient pressure of a microbubble increases due to a nearby or surrounding bubble cloud collapse [15].

**Fig. 1:**
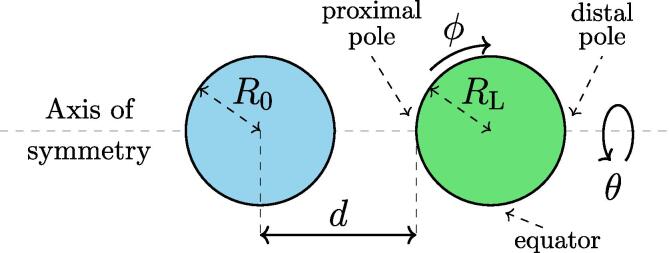
A schematic representation of the considered setup – an initially stable bubble with radius $R_{0}$ (left) in vicinity of a freely submerged spherical liposome with radius $R_{L}$ (right). In addition, the main regions of the liposome’s envelope are also marked: proximal and distal pole (also tip, proximal and distal refer to the position in regard to the bubble) and the equator.

A scheme of the considered phenomenon is shown in Fig. 1, where a non-dimensional stand-off distance parameter δ is defined as $\delta =d/R_{\max}$ (also commonly recognized as γ in studies of near-wall bubble collapse), which is consistent with previous investigations of Le Gac et al. [23]. Bilayer’s local element coordinate system is defined in accordance with here shown coordinates ϕ and θ, which follow its local deformations through time. Coordinate ϕ follows the tangential direction of the envelope in the here considered plane, whereas θ marks the direction of revolution about the axis of symmetry.

Results are reported for twelve cases of bubble-liposome interaction, corresponding to the following values of bubble-liposome distance parameter: δ = 1.15, 1.2, 1.3, 1.4, 1.5, 1.6, 1.7, 1.75, 2, 2.25, 2.5, and 3. Additionally, cases with δ between 1 and 1.15 were also attempted, but resulted in termination even before the first bubble collapse, due to severe wrinkling and deformations of the bilayer, which led to numerical instabilities and a failure in convergence of the structural solver. Surface tension and viscosity of water were set to $\gamma_{w}=72.8\times 10^{-2}$ N/m and $\mu_{w}=10^{-3}$ Pa·s, whereas viscosity of air is also considered with $\mu_{g}=1.8\times 10^{-5}$ Pa·s. Thermal conductivity of water and air are considered as $k_{w}=0.6$ W/mK and $k_{g}=0.0242$ W/mK, respectively. Boundary conditions at the end of the computational domain were set to wave non-reflecting pressure outlet with a static pressure of $10^{7}$ Pa, temperature of 20°C, and volume fraction of water $\alpha_{w}$ set to unity. No slip boundary condition is considered at the vesicle’s shell.

**Fig. 2:**
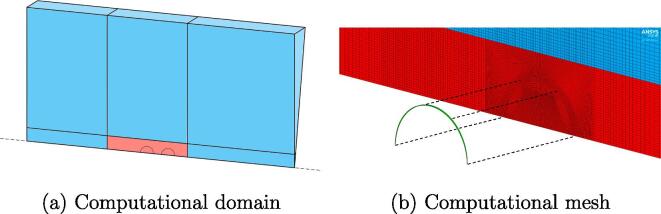
(a) A schematic representation of the utilized wedge geometry and the whole fluid computational domain. The wedge revolves around the axis of symmetry (dashed black line) with the thickness of one computational cell. The fluid domain is divided into two sections: a static mesh region (blue) and dynamic mesh smoothing region (red). Please note that the dimensions of the computational domain on are not directly proportional to the actual geometry for the sake of figure readability. (b) The computational mesh in direct vicinity of the FSI region showing mesh of the fluid domain (blue and red fill) and the solid domain (green fill). Please note that only a fraction of computational cells is shown and the solid domain is offset along the black dashed lines for the sake of visibility.

All simulations are carried out for an axisymmetric case on a wedge geometry, which revolves around the axis of symmetry with the thickness of one computational cell (see Fig. 2 (a)). Computational mesh for the fluid domain consists of orthogonal cells with a constant resolution of 200 cells per initial bubble radius $R_{0}$ in the region of bubble-liposome interaction and gradually coarsened cells with the distance towards the domain’s edge, as can be seen in Fig. 2 (b). This spatial resolution was chosen based on the results of the grid convergence analysis (see Section 3) and the available computational resources. Non-orthogonal quadrilateral cells were used in the direct vicinity and within the liposome’s shell to transition between the spherical envelope and the orthogonal cells in the rest of the computational domain. The fluid domain can be further separated into two zones: a static mesh region and a dynamic mesh smoothing region. Numerical grid in the latter region is dynamically adapted to the bilayer’s movement during each FSI coupling iteration step, as described in Section 2. Multiple values of the spring constant factor were tested during the model setup. In the end, the value of 0 was used for all simulations in order to preserve the quality of the mesh in the direct vicinity of the bubble and liposome. In this way the displacements of the coupling interface, i.e., the liposome’s envelope, were absorbed by larger cells at the edge of the dynamic mesh smoothing region.

The shell itself was geometrically defined with a surface through its mid-plane, which in the present case resembles a thin slice of a sphere with radius of 1 μm. It was discretized with quadrilateral second order shell elements with four in-plane integration points (element SHELL281) in the structural solver and was meshed to be conformal with the mesh of the fluid–solid interface in the fluid domain. Two triangular elements were also used, one at each pole, where the FSI interface meets the axis of symmetry. The total number of computational cells in the fluid domain ranged between 330 and 450 thousand, with 190 to 275 thousand of them being included in the dynamic remeshing process. A constant computational time step was set to 4 ps, which resulted in 6250 time steps for each case. This time step was chosen with respect to the preliminary simulations, from which the discretization errors were estimated. The corresponding results are given in the beginning of Section 3.

Approximately four coupling iterations per time step were performed on average to reach data transfer convergence target of $10^{-3}$. Convergence criteria for the fluid dynamics solver were set with the values of scaled residuals for continuity and momentum equations to $10^{-6}$, and energy equation to $10^{-9}$. In addition, custom convergence criteria were also set to $10^{-6}$ for bubble radius, along with the integrals of pressure and shear stresses over the coupling interface, i.e., both sides of the liposome’s envelope. Convergence criteria in the structural solver were set as default vector norm checks ($L^{2}$ norm for force, moment, rotation and infinity norm for displacement) with the specified tolerance of $10^{-6}$. Computational times varied from 7 to 11 days per case, with each being computed on a 24 core HPC cluster node. Cases with larger values of δ required longer computational times due to the larger extent of their mesh adaptation region in the fluid domain, which turned out to be the limiting factor for the use of even finer computational meshes without increasing the utilized processing power.

## 3 Results

In this section, we address the key topic of the present study – a bubble-liposome interaction on a micro scale. The results cover the effects of a nearby liposome on bubble collapse dynamics and vice versa. First, we present the results of preliminary simulations, from which the finally considered spatio-temporal resolution was chosen. Preliminary results correspond to the case with δ=1.2 and are given in Table 1. They include the estimation of discretization errors and Richardson extrapolation of the normalized minimum bubble radius $R_{\min}/R_{0}$ and peak membrane $\varepsilon_{\max}^{m}$ and bending strains $\varepsilon_{\max}^{\mathrm{tb}}$ of the bilayer. To be more thorough, $\varepsilon_{\max}^{m}$ stands for overall peak strains in the middle throughout the bilayer thickness, whereas $\varepsilon_{\max}^{\mathrm{tb}}$ represents overall peak strains in the envelope at its top/bottom plane. Midpoint strains $\varepsilon^{m}$ can be understood as a measure of bilayer stretching, while strains at the top or bottom of the envelope $\varepsilon^{\mathrm{tb}}$ point to the magnitude of bilayer bending. When $\varepsilon^{m}\approx \varepsilon^{\mathrm{tb}}$, bilayer bending is negligible and lateral stretching can be understood as the main driver of liposome’s deformation.

**Table 1:** Estimation of discretization errors and Richardson extrapolation of the normalized minimum bubble radius $R_{\min}/R_{0}$ and peak membrane $\varepsilon_{\max}^{m}$ and bending strains $\varepsilon_{\max}^{\mathrm{tb}}$ of the bilayer for the case with δ=1.2. The results in the top row correspond to the finally chosen spatio-temporal resolution, whereas the rest serve as a means to estimate the magnitude of discretization errors.

Spatial resolution [nm]	Temporal resolution [ps]	$R_{\min}/R_{0}$ [–]	Estimated error [%]	$\varepsilon_{\max}^{m}$ [–]	Estimated error [%]	$\varepsilon_{\max}^{\mathrm{tb}}$ [–]	Estimated error [%]
5	4	0.114	−5.0	0.123	0.18	0.314	−0.11
20	8	0.099	−18	0.127	3.3	0.264	−16
10	4	0.108	−9.9	0.124	0.75	0.314	−0.15
5	2	0.116	−3.6	0.123	0.17	0.315	$-1.4\times 10^{-3}$
1/∞	1/∞	$0.120^{*}$		$0.123^{**}$		$0.315^{**}$	

∗ Based on the estimation of discretization errors from Zevnik and Dular [30].

∗∗ Estimated values according to the Richardson’s extrapolation of the obtained results.

The results show convergent behavior towards the grid-independent solution, however it is clear that the rate of convergence is much higher for the bilayer’s response, which implies that the driving process of bubble dynamics primarily determines the required spatio-temporal resolution in the present case. As the peak velocities of the bubble’s wall reach the order of ~500 m/s, the finally chosen resolution of Δx=5nm and Δt=4ps ensures that the maximum Courant number in the fluid domain will not exceed 0.4 at any point of the simulation.

**Fig. 3:**
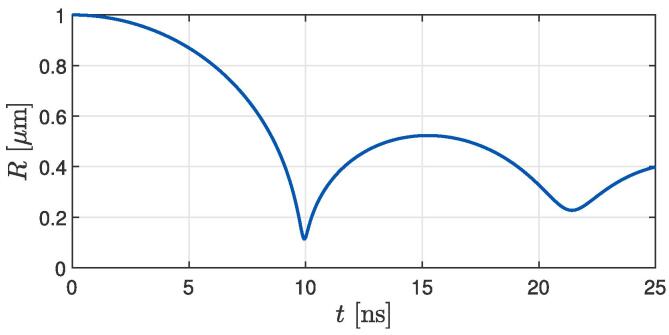
The obtained temporal progression of bubble radius *R* for the case with δ=1.15. The curves from all other cases overlay the one shown here, which indicates that the presence of a nearby liposome does not affect the dynamics of an unattached bubble with δ⩾1.15.

We continue with the effects of a nearby liposome on bubble collapse dynamics. The obtained results show spherical collapses and rebounds of bubbles, which implies that the presence of a nearby liposome does not significantly affect the dynamics of an unattached bubble with δ⩾1.15 on the considered spatial scale of ~1 μm. This is consistent with the results of our previous study [30], where the presence of a similarly sized rigid spherical particle had already a relatively small effect on the dynamics of nearby bubbles with δ>1. Among all the cases, the first and second bubble collapses occur at the time of $t_{c,1}=9.94$ ns and $t_{c,2}=21.4$ ns, respectively. The corresponding bubble radii are $R_{\min,1}=0.114$ μm and $R_{\min,2}=0.227$ μm, whereas the peak bubble rebound is achieved at t=15.2 ns with R=0.523 μm (see Fig. 3). The obtained radii $R_{\min,1}$ coincide with the value for a spherically symmetric bubble collapse, from which we can estimate the discretization error of $R_{\min,1}$ and the corresponding peak pressure values of 1.32 GPa to be in the order of 5% (see Table 1 in [30]).

**Fig. 4:**
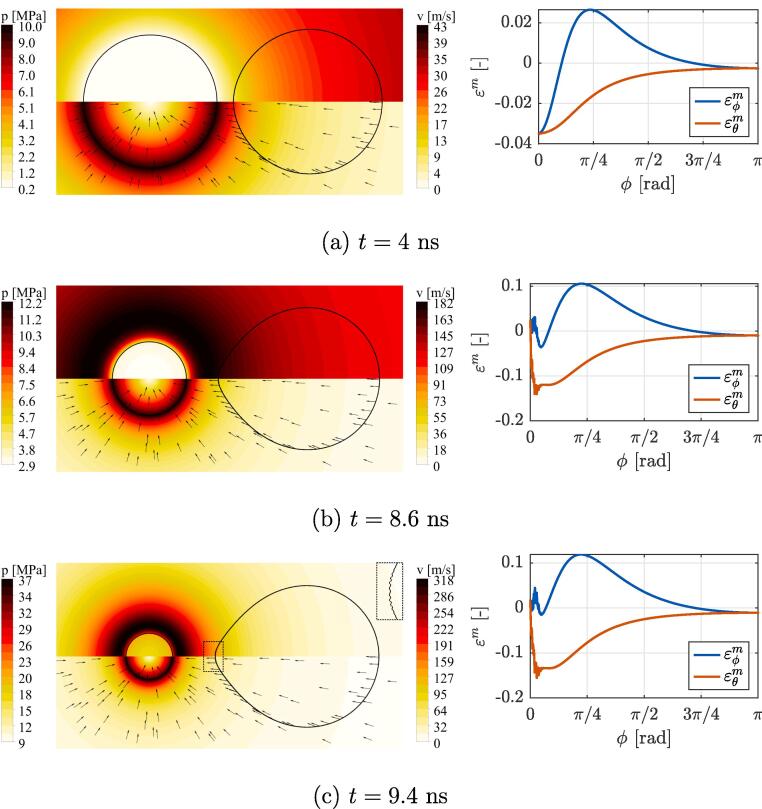
A more detailed insight into the bubble-liposome interaction for the case with δ=1.2, at the time (a) before the onset of bilayer wrinkling, (b) when bubble reaches half of its initial size, and (c) during the bubble collapse, when peak bilayer wrinkling occurs (magnified envelope showing wrinkling of the proximal liposome tip is given in the top right corner of the corresponding contour plot). Left column shows the contours of the pressure (top) and velocity (bottom) fields in the fluid along with the bubble wall (left) and liposome’s envelope (right). The right column shows the spatial distribution of envelope’s midpoint strains $\varepsilon_{\phi }^{m}$ and $\varepsilon_{\theta }^{m}$ in local directions of ϕ and θ, that also correspond to both principal directions.

**Fig. 5:**
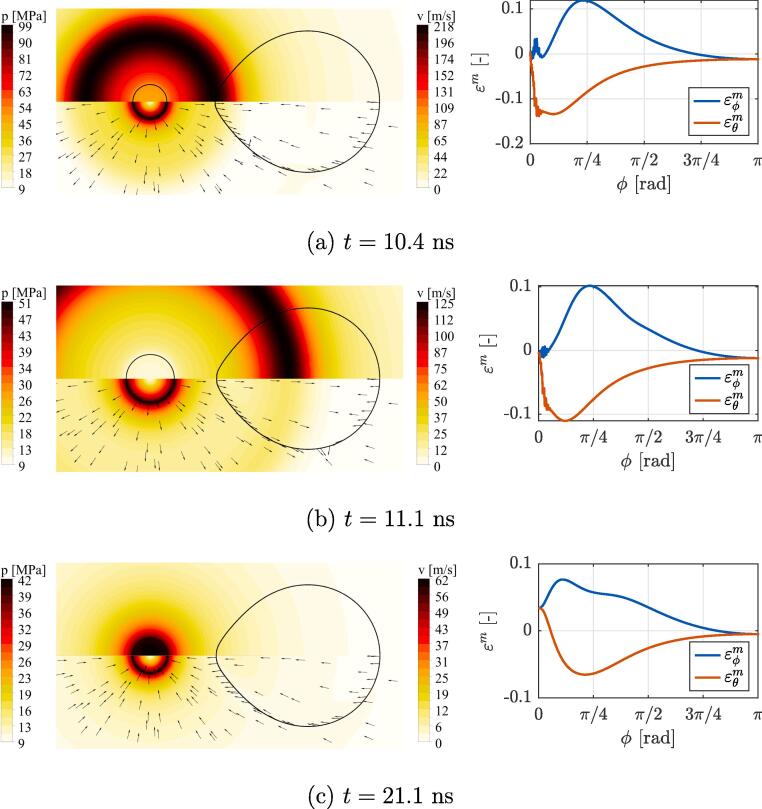
A more detailed insight into the bubble-liposome interaction for the case with δ=1.2, at the time (a) shortly after the bubble collapse, when shock wave reaches the liposome, (b) during shock wave propagation through the vesicle, and (c) during the second bubble collapse. Left column shows the contours of the pressure (top) and velocity (bottom) fields in the fluid along with the bubble wall (left) and liposome’s envelope (right). The right column shows the spatial distribution of envelope’s midpoint strains $\varepsilon_{\phi }^{m}$ and $\varepsilon_{\theta }^{m}$ in local directions of ϕ and θ, that also correspond to both principal directions.

A more detailed insight into bubble-liposome interaction for the case with δ=1.2 is given in Fig. 4, Fig. 5. Here, the left column shows the contours of the pressure (top) and velocity (bottom) fields in the fluid along with the bubble wall (left) and liposome’s envelope (right). The right column shows the corresponding spatial distribution of liposome shell midpont strains $\varepsilon_{\phi }^{m}$ and $\varepsilon_{\theta }^{m}$ with respect to the circumferential direction ϕ (see Fig. 1). The values of ϕ range between 0 and π, which correspond to the proximal and distal pole (also tip) of the liposome, whereas its equator lies at π/2. The term waist refers to the central area of the liposome near the equator. Both local directions of ϕ and θ coincide with both principal directions, i.e. the directions where the normal strain vectors are maximized. To be more thorough, $\varepsilon^{m}$ stands for strains in the middle throughout the bilayer thickness and in the direction marked by the subscript, e.g. $\varepsilon_{\theta }^{m}(\pi /2)$ corresponds to midpoint strain in the local element direction of θ at the location of ϕ=π/2.

Fig. 4 (a) shows a state at the time of 4 ns. Due to a sudden increase of ambient pressure the bubble starts to shrink, which results in a sink-type velocity field in the ambient liquid. A relatively continuous pressure and velocity fields can be observed in the direct vicinity of the envelope and the boundary layer can be barely identified, as the vesicle moves and deforms with the surrounding liquid. Since velocity field decays approximately with the square of a distance from the bubble wall, a nearby vesicle is exposed to a velocity gradient field which induces spatially uneven envelope stretching in the axial direction and contraction perpendicularly to it. The latter causes compressive strains $\varepsilon_{\theta }^{m}$ throughout the whole envelope, with the peak at the proximal pole ($\varepsilon_{\theta }^{m}=-0.035$ at ϕ=0). On the other hand, three zones can be identified for strains $\varepsilon_{\phi }^{m}$ in the tangential direction ϕ - a compressive zone at both poles and a tensile zone at the waist.

**Fig. 6:**
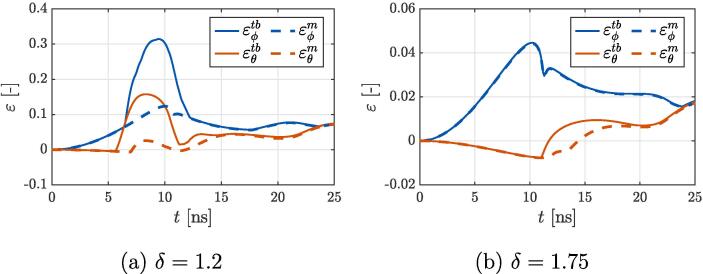
Temporal progression of peak shell strains at midpoint $\varepsilon^{m}$ and top/bottom $\varepsilon^{\mathrm{tb}}$ for the case with (a) δ=1.2 and (b) δ=1.75. The directions of both principal shell strains $\varepsilon_{1}$ and $\varepsilon_{2}$ correspond to the local element directions of ϕ and θ, respectively.

As the bilayer at both poles experiences lateral compression, this can lead to buckling related instabilities in form of local bilayer wrinkling. We use the following first-order approximation to estimate the critical loads for the onset of buckling in thin elastic spherical shells [60]: $p_{\mathrm{cr}}=2\overset{‾}{E}/\sqrt{3(1-\nu^{2})}{\left(\tau_{0}/R_{L}\right)}^{2}$, from which the critical strain can be derived as $\varepsilon_{\mathrm{cr}}\approx (\nu -1)/\sqrt{3(1-\nu^{2})}\left(\tau_{0}/R_{L}\right)$. With $\nu =0.485,\tau_{0}=4$ nm, and $R_{L}=1\mu$m, the critical strain is approximated at $\varepsilon_{\mathrm{cr}}=-1.4\times 10^{-3}$. One can notice, that compressive strains at the proximal pole ($\varepsilon_{\phi }^{m}\approx \varepsilon_{\theta }^{m}=-0.035$) are far beyond the estimated critical value of $\varepsilon_{\mathrm{cr}}=-1.4\times 10^{-3}$, which results in a gradual development of wrinkles through time. This can be also seen in Fig. 6 (a), which shows the temporal progression of peak shell strains at midpoint $\varepsilon^{m}$ and top/bottom $\varepsilon^{\mathrm{tb}}$ through its thickness. The onset of wrinkling can be identified as a sudden split between curves $\varepsilon^{m}$ and $\varepsilon^{\mathrm{tb}}$ at the time around 6 ns, which marks the occurrence of severe bilayer bending.

Fig. 4 (b) shows the state of the system at 8.6 ns, when the bubble shrinks to half of its initial size. The pressure and velocity fields still show a strong resemblance to ones in the case of an unbounded bubble with the development of a ridge-like pressure field just outside the bubble wall. The only directly visible disturbance can be observed at the proximal pole of the vesicle, where the pressure field is locally altered due to changes in the bilayer curvature and the occurrence of wrinkling.The latter also affects the spatial distribution of midpoint strains, which exhibit strong oscillations at the proximal pole. Regardless of that, the vesicle’s waist continues to experience severe stretching in the circumferential direction with the peak $\varepsilon_{\phi }^{m}$ of 0.1 appearing near the point at ϕ=π/4. Similar strain distribution can be seen at the time of 9.4 ns (Fig. 4 (c)), when overall maximum strains in the envelope occur at the proximal pole. This can be also seen on Fig. 6 (a) as a peak of $\varepsilon_{\phi }^{\mathrm{tb}}=0.31$, which exceeds peak midpoint strains $\varepsilon_{\phi }^{m}$ by almost threefold. Again, this indicates the occurrence of severe local wrinkling of the bilayer, which can be also seen in the magnified box in the top right corner of the attached contour plots. Please note, that digital magnification of subfigure (c) is required to observe wrinkling in the non-magnified instance of the proximal liposome tip due to the small wavelength of ~25 nm.

During the bubble collapse at 9.94 ns, both internal bubble pressure and temperature locally reach their peak values in the order of 1.3 GPa and 3500 K. After the peak collapse point is reached, the bubble rebounds and starts to expand. Additionally, a shock wave is emitted into the surrounding liquid and overall peak liposome stretching at the waist with $\varepsilon_{\phi }^{m}=0.12$ is reached. Peak vesicle stretching also coincides with peak displacement of the proximal tip, which reaches its maximum just before the shock wave impact. As the shock wave propagates through the ambient liquid it attenuates with approximately the inverse of the distance from the bubble center (~1/r) and changes the velocity field to the source-like type. It reaches the proximal liposome tip at the time of 10.4 ns with the magnitude of 100 MPa (Fig. 5 (a)), which is also accompanied with a sudden change in the direction the tip’s movement. The magnitude of shock wave front attenuation is perhaps the most clear from the comparison between subfigures (a) and (b), where a twofold decrease of its magnitude can be observed upon the propagation of less than a half of the liposome’s length. Neither of the evaluated cases points towards a reflection of the pressure wave from the liposome’s envelope, which can be explained by the fact, that their envelope is extremely compliant and has almost identical density to water. Furthermore, the liposome’s interior consists of water, which also plays an important role in interaction with pressure waves. Overall, the difference in acoustic impedance between water and liposomes seems to be negligible to cause noticeable disturbance in shock wave propagation.

As the shock wave propagates through the envelope (Fig. 5 (b)) it causes a second local maximum of liposome stretching at the waist. This can be seen in Fig. 6, that shows temporal progression of peak $\varepsilon_{\phi }^{m}$ at the time of ≈ 11 and 12 ns for cases (a) δ=1.2 and (b) δ=1.75, respectively. Additionally, on subfigure (a) one can also notice a drop in top/bottom shell strains $\varepsilon^{\mathrm{tb}}$ after bubble collapses, which implies a gradual smoothening of the wrinkles. This is to be expected, since after shock wave propagation the envelope’s movement is reversed towards its initial undeformed state. Consequently, compressive strains at the proximal tip slowly decay, indicating a transition to a tensile phase. The expanding bubble reaches the maximum size of the rebound phase with R=0.523 μm at t=15.2 ns, after the pressure wave has already propagated past the vesicle. This is soon followed by the less intense second bubble collapse at $t_{c,2}=21.4$ ns with $R_{\min,2}=0.227$ μm. The second collapse also results in the emission of a shock wave, although weaker in magnitude and more spatially flattened due to the slower process of the collapse. After all, a large part of the bubble’s energy has been already lost with the emission of the first shock wave.

Fig. 5 (c) shows a state during the second bubble collapse. Here, a boundary layer at the vesicle’s envelope is more prominent because the elastic effects of the bilayer come to effect after the first collapse. This can be also seen from the directions of velocity vectors at the bilayer, which are not directly aligned with the sink-type surrounding flow, implying the presence of vesicle’s own elastic oscillations. Looking at the corresponding spatial distribution of shell midpoint strains, we can observe that the proximal tip is under uniform stretching with $\varepsilon^{m}=0.034$. Time-wise, it’s peak magnitudes occur during the time of second bubble contraction, which corresponds to local maximums of $\varepsilon_{\theta }^{m}$ (Fig. 6) at 16.2 and 18.1 ns for cases (a) δ=1.2 and (b) δ=1.75, respectively. On the other hand, the distal pole of the vesicle remains in uniform compression for the whole simulated time of 25 ns and the values for the most part exceed the approximated critical value of $\varepsilon_{\mathrm{cr}}=-1.4\times 10^{-3}$. Although we noticed minor bending of this region upon a more thorough inspection of the results, it remains invisible to the naked eye and negligible in terms of through-thickness distribution of shell strains.

A comparison between both cases in Fig. 6 reveals a relatively similar temporal progression of peak midpoint shell strains $\varepsilon^{m}$ between cases with δ=1.2 and 1.75. Peak strains in tangential direction $\varepsilon_{\phi }^{m}$ increase with time and reach their maximum around the time of the first bubble collapse. Shortly after, another lesser local maximum can be observed, which is related to shock wave propagation through the liposome. Both maximums occur at the liposome’s waist, at the point with the initial coordinate of ϕ≈π/4. On the other hand, the envelope is for the most part compressed in direction of θ (revolution about the axis of symmetry). One exception is the emergence of a tensile zone at the proximal pole after the bubble rebounds. Therefore peak values of $\varepsilon_{\theta }^{m}$ progressively increase in an oscillating manner and merge with $\varepsilon_{\phi }^{m}$ after the second bubble collapse, which results in uniform lateral stretching of the proximal tip. A local maximum of peak $\varepsilon_{\theta }^{m}$ can be identified for all cases, at the times between 15 and 20 ns. On the contrary, peak top/bottom shell strains $\varepsilon^{\mathrm{tb}}$ show less similarities between the cases. Generally, they can be classified into two groups, depending on the development of bilayer wrinkling. From small enough values of δ (subfigure (a)) a noticeable split between curves $\varepsilon^{\mathrm{tb}}$ and $\varepsilon^{m}$ can be seen around the time of 6 ns. As already mentioned before, this marks the onset of severe bilayer bending and wrinkling, which reaches its maximum around the time of the first bubble collapse. Later, when the bubble rebounds and the velocity field is reversed to a source-type, the wrinkles gradually smoothen out. This can be seen as a decrease in $\varepsilon^{\mathrm{tb}}$ and later its merging with the curve $\varepsilon^{m}$. With larger values of δ (subfigure (b)) liposome wrinkling becomes less intense and progressively vanishes.

**Fig. 7:**
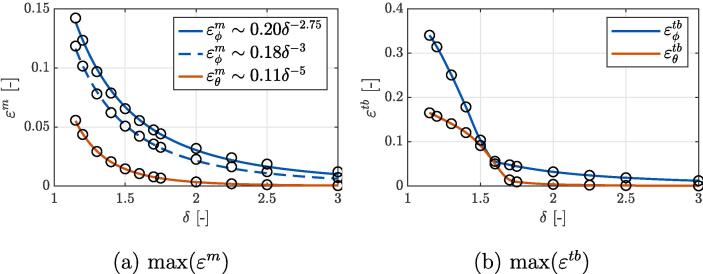
Peak (a) shell midpoint strains $\varepsilon^{m}$ and (b) shell strains at top/bottom $\varepsilon^{\mathrm{tb}}$ in both local directions ϕ and θ, marked by the subscript. Values are given in relation to the initial bubble-liposome distance δ. Curves in subfigure (a) represent the power law fits ($R^{2}>0.995$) of the obtained results from the simulations (hollow circles).

All mentioned local maximums of envelope strains are given in Fig. 7 in relation to the initial bubble-liposome distance δ. In subfigure (a), both curves of $\varepsilon_{\phi }^{m}$ correspond to peak bilayer extension at the waist, which occurs at the first bubble collapse (solid line) and shortly after, when a shock wave travels through the liposome (dashed line). One can notice, that the dashed curve consistently stays below the solid line, which implies that in the present range of parameter δ, overall peak liposome stretching occurs at it’s waist during the time of the first bubble collapse. The third curve, $\varepsilon_{\theta }^{m}$, corresponds to peak bilayer extension at the proximal tip, which occurs during the second bubble contraction. For all three sets, the obtained results from the simulations (hollow circles) monotonously descend according to the power functions ($R^{2}>0.995$) with exponents of −2.75, −3, and −5. A similar trend can be seen for cases with δ>1.75 in subfigure (b), which shows overall maximum values of top/bottom shell strains $\varepsilon^{\mathrm{tb}}$ in both principal directions. Here, we can clearly notice the development of bilayer wrinkling at the proximal tip for cases with δ<1.75, where a power law-like behavior transitions to a polynomial one, which surpasses the corresponding midpoint strains by a much as twofold. Although the temporal onset of peak strains $\varepsilon^{\mathrm{tb}}$ varies with δ it generally occurs around the time of the first bubble collapse.

The magnitude of bubble’s liposome destruction potential can be estimated upon comparison of the obtained results with both previously determined bilayer rupture thresholds $\varepsilon_{p}^{*}=0.45$ and $\varepsilon_{s}^{*}=0.035$ (see Section 2.3 and Appendix B). First, we can conclude that none of the numerically evaluated cases with a bubble-liposome distance parameter δ between 1.15 and 3 exceeds the primary failure criterion $\varepsilon_{p}^{*}=0.45$, which is related to the creation of a defect and a subsequent membrane rupture due to heavy lateral loading. On the other hand, for small enough values of δ, we can notice that both liposome stretching ($\varepsilon^{m}$) and wrinkling ($\varepsilon^{\mathrm{tb}}$) can surpass the secondary failure criterion $\varepsilon_{s}^{*}=0.035$ by as much as ten-fold. In the case of pre-existing pores in the bilayer can we therefore expect the damage to occur at the waist of the vesicle for δ below 1.9 and at the vesicle’s pole for δ under 1.25. Additionally, bilayer wrinkling at the nearer pole could also aggravate existing defects in cases with δ⩽1.6.

## 4 Discussion

**Fig. 8:**
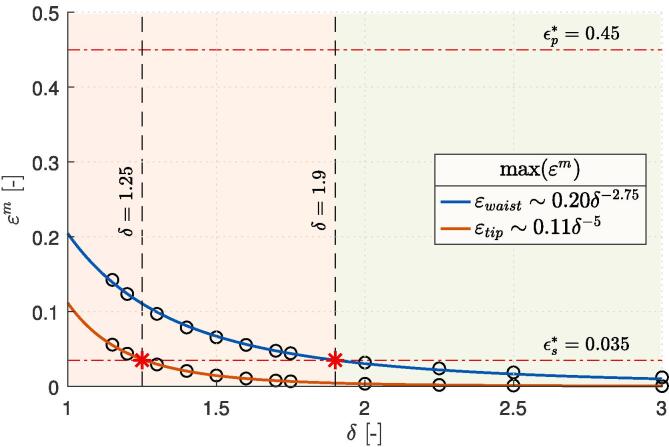
Peak values of local bilayer extension at the vesicle’s tip $\varepsilon_{\mathrm{tip}}$ and waist $\varepsilon_{\mathrm{waist}}$ in relation to the initial bubble-liposome stand-off distance δ. Curves represent the power law fits of the obtained results from the simulations (hollow circles) with $R^{2}>0.995$.

In this section, the obtained results are further discussed and extrapolated with respect to liposome destruction by hydrodynamic cavitation. First, we extrapolate the obtained peak strains related to local bilayer stretching at the proximal vesicle’s tip $\varepsilon_{\mathrm{tip}}$ and waist $\varepsilon_{\mathrm{waist}}$ (see Fig. 8), which correspond to $\varepsilon_{\theta }^{m}$ and $\varepsilon_{\phi }^{m}$ in Fig. 7 (a). This is done to estimate the needed value of non-dimensional bubble-liposome standoff distance δ to achieve severe bilayer stretching beyond its primary failure criterion $\varepsilon_{p}^{*}=0.45$ and thus vesicle’s destruction on a nanosecond temporal scale. Both extrapolations (solid curves) are based on the obtained results from the simulations (hollow circles) and follow power functions with exponents of −2.75 ($R^{2}=0.998$) and −5 ($R^{2}>0.999$) for peak liposome strains at its waist and tip, respectively.

Both extrapolated curves meet the value of $\varepsilon_{p}^{*}=0.45$ at δ~0.75, which is in remarkably good agreement with the values reported from previous experimental studies addressing single bubble-cell interaction of Le Gac et al. [23]. They used single laser-induced cavitation microbubbles to porate suspended human promyelocytic leukemia cells and observed cell lysis probability of more than 75% for δ⩽0.75. As already mentioned in Section 1, similar effective distance for cell membrane poration was later also reported by Zhou et al. [22], who acoustically excited single laser-induced microbubbles in vicinity of a Xenopus oocyte. Despite the good match with both mentioned experimental studies, we acknowledge that the actual bubble dynamics and the deformation process of liposomes could be qualitatively different for small values of δ. This also holds for the cases where the bubble undergoes initial expansion before it collapses, which largely depends on the boundary conditions, type of cavitation, size of bubbles, etc. In this scenario, values of δ below 1.0 are not uncommon. For this reason we limit the extrapolation of data to δ>1.0 and conclude that liposomes with equilibrated envelopes, i.e., no pores are present in the bilayer, are not expected to be structurally compromised in cases with δ>1.0, when a nearby collapsing bubble is not in their direct contact. As an approximation, this could be also extended to other liposome-like biological structures, such as single suspended cells (e.g. leukocytes [23], erythrocytes [28]) or cell’s organelles, although a special care would have to be given to the extent of their structural similarity to here considered giant unilamellar DOPC vesicles.

However, from here obtained results we can expect local bilayer rupture in the case of pre-existing semi-stable defects, which could remain from previous mechanical stresses. The limiting values of δ are obtained from the intersections (red asterisk) of both curves representing peak bilayer strains, $\varepsilon_{\mathrm{tip}}$ and $\varepsilon_{\mathrm{waist}}$, with the secondary failure criterion $\varepsilon_{s}^{*}=0.035$ at δ=1.25 and 1.9, respectively. In other words, liposomes are expected to be unaffected for δ>1.9, regardless of the existence of past bilayer defects.

As already mentioned in the end of previous section, bilayer wrinkling at the pole could also aggravate existing defects in cases with δ under 1.6, although it remains unclear whether it could also cause rupture of previously undamaged membranes. The reason for this is, that we were unable to extrapolate the obtained peak bending strains from Fig. 7 (b) below δ=1.15 with a sufficient degree of confidence. After all, a relatively small region of 1.15⩽δ⩽1.5 is available for extrapolation of a polynomial-like trend, which can yield vastly different results. Therefore this remains as one of the challenges for our further investigations, where encountered numerical instabilities in the cases with δ<1.15 should be addressed more in-depth.

Qualitatively, the presented results are also in good agreement with the findings of Marmottant and Hilgenfeldt [20], who experimentally showed that gently oscillating single bubbles excited by an ultrasound can already result in controlled deformation and lysis of DOPC vesicles of similar sizes. In their later work, [21] derived analytical predictions of vesicle shape progression and found two possible modes of liposome damage: a) pore formation at vesicle’s waist in the case of sufficiently large shear rates and b) liposome buckling at the poles in the case of sufficient liposome elongation. These predictions are further supported by the present numerical investigations. In addition to this, we also identified a third relevant mode of liposome damage in the case of an inertial bubble collapse – membrane poration at the liposome’s tip, which could occur during the contraction phase of a rebounding bubble.

At this point, it might be worth mentioning again, that the reported values of effective distances for liposome poration are given solely for here considered mechanical effects that result from a single bubble collapse. It is known, that strong bubble collapses are also linked to chemical effects, which are caused by the homolysis of vaporous water molecules. This leads to formation of reactive oxygen species, namely ^.^OH and ^.^H [16]. The formation of reactive oxigen species can affect biological structures chemically, via oxidation, although the effective distances for the poration of various cells and bio membranes in the case of a single bubble collapse are not yet known [10].

**Fig. 9:**
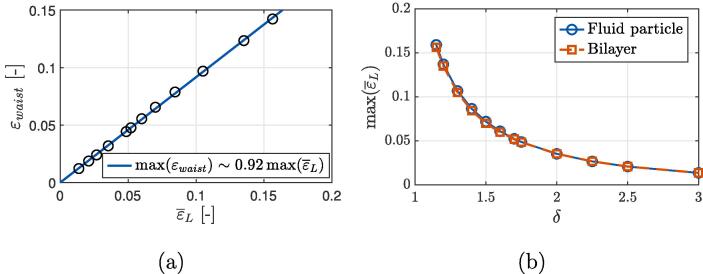
(a) A linear relation ($R^{2}$> 0.999) between peak liposome length strains ${\overset{‾}{\varepsilon }}_{L}$ and peak local bilayer strains at the waist $\varepsilon_{\mathrm{waist}}$ among all evaluated cases. (b) Peak length strains according to surrogate fluid particle movement simulations and actual FSI simulations of bubble-liposome interaction show a good level of agreement with maximum discrepancy of less than 3%.

Since we are addressing biological structures on a scale length of a few micrometers, liposome’s overall length extension ${\overset{‾}{\varepsilon }}_{L}=(L-L_{0})/L_{0}$, i.e. relative change in the distance between both poles, might be a more useful measure of it’s deformation and stretching as it could be also experimentally obtained. Upon evaluation of maximum length strains ${\overset{‾}{\varepsilon }}_{L}$ we noticed, that their peaks temporally coincide with overall peaks in local bilayer stretching at the waist $\varepsilon_{\mathrm{waist}}$, which occurs shortly after the first bubble collapse, just before the emitted shock wave reached the vesicle. Although this might not come as a surprise, we find the fact that they also show a clear linear relation of $\max(\varepsilon_{\mathrm{waist}})\sim 0.92\max({\overset{‾}{\varepsilon }}_{L})$ ($R^{2}>0.999$) between all evaluated values of δ quite interesting (Fig. 9 (a)). After all, this shows that peak local bilayer’s extension at the waist is of a similar magnitude to vesicle’s overall extension between both poles and that their dependence does not change with bubble-liposome distance. However, we speculate that the slope of linear relationship is dependent on other geometric parameters, such as liposome-bubble size ratio. This can be explained by the velocity gradient distribution, which drives the envelope stretching. For an incompressible case of a collapsing spherical bubble, velocity gradient decays with a cube of the radial distance from the bubble’s wall, which implies that larger bubbles will cause a more even spatial distribution of bilayer stretching, whereas the effects of smaller bubbles will be more locally limited to the liposome’s proximal pole.

At this point, it might be worth mentioning again, that we are here considering an initially stable microbubble in vicinity of an undeformed DOPC liposome of a similar size. Vesicle stretching and deformation is driven by an inertially collapsing bubble, which occurs due to a sudden increase in ambient pressure, e.g. collapse of a nearby bubble cluster results in emission of a shock wave, which propagates past the bubble-liposome pair and causes the bubble to collapse. Nonetheless, when one considers the phenomenon of hydrodynamic cavitation, many other scenarios of bubble behavior are to be expected, depending on the development of the cavitating flow. For example, many bubbles experience significant growth due to an initial ambient pressure decrease and collapse only after they have reached a region of increased ambient pressure. Maximal size of those bubbles can thus exceed the size of nearby liposomes by a few orders of magnitude. Additionally, more than one bubble can be present in the vesicle’s vicinity, which could amplify or even dampen the loads exerted on the bilayer. Certainly, a plethora of questions arises when one considers the whole range of possible scenarios. Although advanced numerical simulations can prove as an invaluable tool, especially when considering phenomena on very small spatial and temporal scales, their feasibility can be limited due to numerous constraints. For example, here utilized methodology is currently limited to the axially symmetric scenarios, as a full 3D model would be simply too computationally demanding. We face similar problems when considering much larger bubbles ($R_{\max}\gg R_{L}$) in vicinity of a micrometer-sized vesicle, as the needed spatial resolution does not scale with the bubble’s size due to a nearby liposome. After all, as already mentioned in Section 2.5, computational times for presently considered scenarios already amounted to between 7 and 11 days per case, with each being computed on a 24 core HPC cluster node.

Having said that, we are still able to utilize much simpler and computationally less demanding models to estimate whether the collapse of larger bubbles also carries a potential for liposome’s destruction. To achieve this, we consider the observation from the previous section, that the liposomes’s envelope movement closely follows the movement of the surrounding liquid until the first collapse, which implies its inertial movement with negligible elastic effects (witch an exception of bilayer wrinkling at the proximal pole). The reason for fluid-like behavior of the vesicle can be found in a relatively high compliance of the bilayer, which is due to its inherent material characteristics, small thickness (~4 nm), and similar density to water. As peak magnitudes of liposome stretching $\varepsilon_{\mathrm{waist}}$ clearly correlate with peak length strains ${\overset{‾}{\varepsilon }}_{L}$ (Fig. 9 (a)) and both occur at the time of the first bubble collapse, we can thus predict peak values of ${\overset{‾}{\varepsilon }}_{L}$ by only resolving pathlines of two fluid particles corresponding to the location of both liposome’s poles. Through this we can omit the modeling of a full FSI system and only resolve a simpler case of spherical bubble collapse, since the presence of a nearby liposome does not seem to significantly affect the dynamics of a collapsing bubble of a similar size (see Section 3). This significantly simplifies the considered problem at hand and shortens the required computational times per case by more than a ten-fold.

A comparison of the obtained results between the actual FSI and surrogate simulations is given in Fig. 9 (b). A very good agreement between both curves can be observed, with maximum relative discrepancy of less than 3%, which occurs at the smallest considered value of δ=1.15. For the most part, we attribute the difference to the emergence of bilayer wrinkling, which cannot be predicted by only resolving fluid flow. Further simplifications were attempted by resolving the Rayleigh-Plesset equation, without consideration of the water’s compressibility and emission of shock waves. This even further reduced the required computational times to a matter of seconds per case. Although it resulted in surprisingly good agreement with the obtained results from FSI simulations for δ⩽1.5 (discrepancies within few percents), the relative error began to increase with larger values of δ due to the neglection of compressibility effect in form of continued ”liposome” stretching during the time of shock wave propagation from the collapsing bubble to the liposome’s proximal tip.

**Fig. 10:**
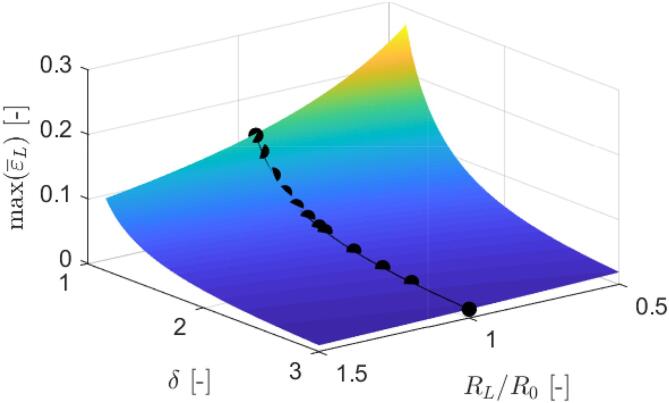
Estimated values of peak liposome length strains ${\overset{‾}{\varepsilon }}_{L}$ with respect to the non-dimensional liposome-bubble stand-off distance δ and their size ratio $R_{L}/R_{0}$, which imply that larger bubbles carry a higher potential for causing stretching-induced liposome destruction. The corresponding results from FSI simulations are included with solid spherical markers.

In order to estimate whether larger bubbles are potentially more harmful in terms of induced peak liposome stretching, we performed a set of surrogate simulations with accounted compressibility effects for a parameter space of 1.15⩽δ⩽3 and $0.5⩽R_{L}/R_{0}⩽1.5$. Here $R_{L}/R_{0}$ represents the ratio between the initial liposome and bubble radius, respectively. The results are given in Fig. 10, from where we can observe that peak length strains ${\overset{‾}{\varepsilon }}_{L}$ exhibit monotonous and accelerated growth towards the smaller values of both parameters, δ and $R_{L}/R_{0}$. Additionally, the corresponding results from FSI simulations are included with solid spherical markers for reference. From this we speculate that larger bubbles carry a higher potential for causing stretching-induced liposome’s destruction, which will be more thoroughly addressed in the future. This could also explain previously observed efficiency of supercavitation for eradication of bacteria, such as *E. coli*, *L. pneumophila*, and *B. subtilis* [6]. As large cavitation clouds form, shed, and collapse, they exert long lasting velociy gradients on bacterium’s envelope, which causes its stretching that eventually leads to poration and rupture of the inner cell membrane and subsequent cell lysis.

## 5 Conclusions

Hydrodynamic cavitation poses as a promising new method for wastewater treatment as it has been shown to be able to eradicate bacteria, inactivate viruses, and destroy other biological structures, such as liposomes. Although engineers are already commercializing devices that employ cavitation, we are still not able to answer the fundamental question: What precisely are the mechanisms of how bubbles can clean, disinfect, kill bacteria and enhance chemical activity?.

The aim of the present paper was to research the dynamics of a single cavitation microbubble ($R_{\mathrm{eq}}=1\;\mu m$) in vicinity of a DOPC lipid vesicle of a similar size, which allowed for a better explanation of the mechanisms behind the recently observed liposome destruction by the hydrodynamic cavitation treatment [8]. Due to small spatial (~1μm) and temporal (~10 ns) scales of the considered phenomenon a purely numerical approach was used. A coupled fluid–structure interaction model was employed, which considered the influence of liposome’s deformability on the surrounding fluid flow and bubble dynamics, and vice versa. Compressible multiphase flow was resolved using a finite volume/volume of fluid method approach, whereas the liposome’s envelope was modeled as a compliant structure through the finite element method. Simulations were carried out for various cases of bubble-liposome standoff distance δ between 1.15 and 3. The required computational times varied between 4000 and 6500 core-hours, where cases with larger values of δ required longer computational times due to the larger extent of their mesh adaption region in the fluid domain.

Regardless of the nearby liposome, the results show spherical bubble behavior, which points towards the negligible effect of vesicle’s presence on the dynamics of a nearby unattached (δ>1) and similarly sized cavitation bubble. As the bubble collapses due to increase in ambient pressure, vesicle deformation is driven according to the temporal development of the surrounding flow field. Three critical modes of vesicle deformation were identified and temporally placed in relation to their corresponding driving mechanisms: (a) unilateral bilayer stretching at the waist of the liposome during the first bubble collapse and subsequent shock wave propagation, (b) local wrinkling at the tip of the liposome until the bubble rebounds, and (c) bilateral bilayer stretching at the tip of the liposome during the phase of a second bubble contraction. Here, unilateral and bilateral stretching refer to the local in-plane extension of the bilayer in one and both principal directions, respectively.

Based on the obtained results, effective distances for liposome poration and rupture were identified, which are in good agreement with previous bubble-cell interaction studies. Liposomes with equilibrated envelopes, i.e., no pores are present in the bilayer, are not expected to be structurally compromised in cases with δ>1.0, when a nearby collapsing bubble is not in their direct contact. However, the critical dimensionless distance for vesicle poration and rupture is identified at δ=1.9 for the case of an envelope with pre-existing pores. In other words, liposomes are expected to be unaffected for δ>1.9, regardless of the existence of past bilayer defects. Results were further discussed with respect to vesicle destruction by the hydrodynamic cavitation treatment, where the influence of bubble-liposome size ratio was also addressed. A higher potential of larger bubbles for causing stretching-induced liposome destruction was identified, which can be also used to explain previously observed efficiency of supercavitation for eradication of bacteria [6].

## CRediT authorship contribution statement

**Jure Zevnik:** Conceptualization, Formal analysis, Writing - original draft, Writing - review & editing, Visualization. **Matevž Dular:** Conceptualization, Writing - review & editing, Supervision, Funding acquisition.

## CRediT authorship contribution statement

**Jure Zevnik:** Conceptualization, Formal analysis, Writing - original draft, Writing - review & editing, Visualization. **Matevž Dular:** Conceptualization, Writing - review & editing, Supervision, Funding acquisition.

## Declaration of Competing Interest

The authors declare that they have no known competing financial interests or personal relationships that could have appeared to influence the work reported in this paper.

## Figures and Tables

**Fig. 11:**
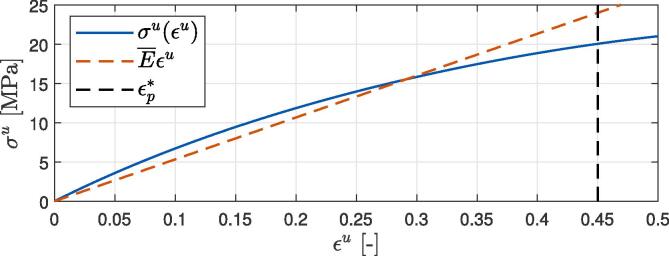
The obtained $\sigma^{u}$-$\varepsilon^{u}$ curve for the DOPC bilayer and the corresponding linearized material model $\overset{‾}{E}{∊}^{u}$.

## Appendix A Determination of DOPC bilayer elastic modulus $\overset{‾}{E}$

Membrane tension in lipid bilayers can be generally written as [61]:

(12) $$\gamma =2\Pi_{0}\left[1-{\left(\frac{a_{0}}{a_{l}}\right)}^{3}\right],$$

where $\Pi_{0}$ is a tension-free surface pressure, which amounts to 50 mN/m for DOPC bilayers. Here, $a_{0}=0.68{\mathrm{nm}}^{2}$ denotes the tension-free area per lipid molecule and $a_{l}$ area per molecule corresponding to a given value of membrane tension γ. In the present case, it is more practical to express membrane tension in relation to areal strain $\varepsilon_{A}=a_{l}/a_{0}-1$:

(13) $$\gamma (\varepsilon_{A})=2\Pi_{0}\left[1-{\left(\frac{1}{1+\varepsilon_{A}}\right)}^{3}\right]\text{.}$$

Membrane tension can be also obtained by integration of average membrane stresses in the lateral direction σ throughout its thickness τ, which yields

(14) $$\gamma =\frac{\left(\sigma_{1}+\sigma_{2}\right)}{2}\tau_{0}(1+\varepsilon_{\tau }),$$

where $\tau_{0}=4$ nm is the initial bilayer thickness and $\varepsilon_{\tau }$ its strain corresponding to $\varepsilon_{A}$. Additionally, $\sigma_{1}$ and $\sigma_{2}$ represent both in-plane principal stresses. For the case of uniaxial stretching, where $\sigma_{1}=\sigma^{u}$ and $\sigma_{2}=0$, the areal strain $\varepsilon_{A}$ and strain throughout the bilayer’s thickness $\varepsilon_{\tau }$ can be written as

(15) $$\begin{array}{c} \varepsilon_{A}^{u}=(1+\varepsilon^{u})(1-\nu \varepsilon^{u})-1\;\mathrm{and}\\ \varepsilon_{\tau }=-\frac{2\nu }{1-\nu }\varepsilon^{u}, \end{array}$$

where ν=0.485 denotes the Poisson’s ratio of the bilayer [47]. Considering this alongside Eq. (13), (14), we can obtain the final relation between linear strain $\varepsilon^{u}$ and the corresponding stress $\sigma^{u}$ for the case an uniaxially loaded bilayer:

(16) $$\sigma^{u}=\frac{4\Pi_{0}}{\tau_{0}(1-\nu \varepsilon^{u})}\left[1-{\left(\frac{1}{\left(1+\varepsilon^{u})(1-\nu \varepsilon^{u}\right)}\right)}^{3}\right]\text{.}$$

The material elastic modulus *E* can be obtained as the initial slope of the obtained $\sigma^{u}-\varepsilon^{u}$ curve, which amounts to 77.3 MPa. This is equivalent to the area stretch modulus $k_{A}=E\tau_{0}/2(1-\nu )=300$ mN/m, which is reported for a full range of accessible tensions in the existing literature and is also applicable to the cases of fast hyper-stretching of the DOPC bilayer [62], [61]. One can also notice that the relation in Eq. (16) shows the inherent material nonlinearity of the DOPC bilayer, which can be seen through stress-softening at large strains.

Interestingly, the obtained relation shows a much lesser magnitude of stress-softening than one might initially expect from Eq. (12). Because of this, the consideration of material as linearly elastic can still provide relevant results if one considers the equivalent elastic modulus $\overset{‾}{E}$ over the whole area of interest on the $\sigma^{u}-\varepsilon^{u}$ curve, bounded by the material failure criterion of $\varepsilon^{*}$ (see Fig. 11):

(17) $$\overset{‾}{E}=\frac{2}{{\varepsilon^{*}}^{2}}\int_{0}^{\varepsilon^{*}}\sigma^{u}(\varepsilon^{u})d\varepsilon^{u}\text{.}$$

Considering the primary failure criterion of $\varepsilon_{p}^{*}=0.45$ (see Appendix B) as the upper bound of $\varepsilon^{*}$, we obtain the equivalent elastic modulus of $\overset{‾}{E}=53.3$ MPa.

## Appendix B Determination of DOPC bilayer material failure criteria

Relevant failure criteria are established based on the critical lateral stresses and the corresponding surface tensions from the existing literature. The reported values are translated to the case of uniaxial loading. This allows for the use of phenomenological failure criteria in a general loading scenario, which does not necessarily result in uniform membrane tension.

### B.1 Primary failure criterion $\varepsilon_{p}^{*}$

According to the primary bilayer failure criterion, bilayer rupture is limited by the creation of a defect due to heavy lateral loading. For an equibiaxial loading case, where $\sigma_{1}=\sigma_{2}$, the critical value for lipid bilayers is in the order of 20 MPa [56], [54], [57]. As defect formation and subsequent membrane rupture is related to the strength of intramolecular forces within the bilayer, we consider the same value of $\sigma_{p}^{*}=20$ MPa as the ultimate tensile strength on a temporal scale of ~10 ns. The corresponding ultimate tensile strain from Eq. (16) amounts to $\varepsilon_{p}^{*}=0.45$.

### B.2 Secondary failure criterion $\varepsilon_{s}^{*}$

In this case, material failure is limited by the expansion of pre-existing meta-stable pores that can emerge from prior mechanical stresses. When pores and defects are already present in a bilayer, much lesser loads are needed to cause their uncontrollable expansion and subsequent membrane rupture. For the case of equibiaxial extension, the critical value is in the order of 5 MPa [56], which corresponds to the linear strain of 3.5%. As material failure is dependent on the expansion of the pore itself, we consider the corresponding strain of $\varepsilon_{s}^{*}=0.035$ as the secondary failure criterion on a temporal scale of ~10 ns. According to Eq. (16) the critical strain corresponds to $\sigma_{s}^{*}=2.57$ MPa.
